# YOLOv8s-BISW a Surface Defect Detection Algorithm for Stainless Steel Pipes

**DOI:** 10.3390/s26113573

**Published:** 2026-06-04

**Authors:** Ziyi Yang, Runwei Gu, Likai Zhu, Xiaocheng Wang, Cheng He, Yujie Wang

**Affiliations:** 1 School of Intelligent Manufacturing and Control Engineering, Shanghai Polytechnic University, Shanghai 201209, China; 20241510058@sspu.edu.cn (Z.Y.); 20251510074@sspu.edu.cn (L.Z.); 20221513066@sspu.edu.cn (X.W.); hecheng@sspu.edu.cn (C.H.); 2 Hudong Heavy Machinery Co., Ltd., Shanghai 200437, China; grw@hhm.com.cn

**Keywords:** stainless steel pipe, surface defect, BiFPN, WIoU, Vision-based inspection

## Abstract

Stainless steel pipes are critical components in industrial systems such as oil and gas transportation and nuclear power cooling. Surface defects can severely degrade their mechanical performance and operational safety. However, existing inspection methods still face challenges including difficult feature extraction, strong reflection interference, and limited accuracy in small-target detection. To address these issues, this paper proposes an improved detection algorithm termed YOLOv8s-BISW (incorporating BiFPN, SGE attention, and WIoU loss), which introduces multidimensional optimizations based on the YOLOv8s baseline. First, an image enhancement module combining Gamma correction and Contrast Limited Adaptive Histogram Equalization (CLAHE) is designed to mitigate uneven illumination and blurred defect imaging. Second, a Bidirectional Feature Pyramid Network (BiFPN) structure is introduced to strengthen multi-scale feature fusion and improve adaptability to defects of different sizes. Meanwhile, a Spatial Group-wise Enhance (SGE) attention module is embedded into the backbone to enhance defect feature representation while suppressing background interference. Furthermore, the Wise Intersection over Union (WIoU) loss function replaces Complete IoU (CIoU) to improve bounding box regression for irregular defects. Experimental results show that the proposed model achieves an mAP of 0.979 on a self-constructed Stainless-steel Tube Flaw (STF) dataset. Compared with the original YOLOv8s, precision, recall, and mAP are improved by 0.007, 0.010, and 0.033, respectively, while the average detection time per image is only 3.7 ms, achieving a favorable balance between accuracy and real-time performance. Compared with mainstream algorithms such as SSD, YOLOv3, and Faster R-CNN, the proposed method demonstrates superior overall performance, providing reliable technical support for automated surface defect detection of stainless steel pipes and offering practical value for intelligent manufacturing quality control.

## 1. Introduction

Stainless steel pipes are widely used in critical industrial applications such as long-distance oil and gas transportation, chemical processing systems, nuclear power cooling circuits, and pipelines in the pharmaceutical and food industries, due to their excellent corrosion resistance, mechanical strength, and manufacturability [1,2]. Under extreme service conditions—for example, deep-sea pipelines subjected to pressures exceeding 10 MPa and corrosive environments, or nuclear cooling pipelines operating at temperatures above 300 °C under radiation exposure—the structural integrity of stainless steel pipes is directly related to operational safety [3,4]. Surface defects not only deteriorate the mechanical properties and corrosion resistance of the material but may also trigger catastrophic failures under coupled high-temperature, high-pressure, and corrosive conditions, posing serious risks to industrial safety, product quality, and the ecological environment.

Early inspection in industrial practice primarily relied on manual visual inspection assisted by simple tools, and in some specific scenarios, contact-based mechanical detection methods were adopted. However, such methods may damage surface coatings and increase the risk of corrosion. Moreover, they are highly dependent on operator experience and are prone to fatigue, with false detection rates reaching 5–10% in complex backgrounds or micro-crack inspection tasks. Their low efficiency further limits their applicability in modern high-throughput production lines.

With the rapid development of machine vision, non-contact inspection systems based on image analysis have been increasingly applied to surface defect detection of steel pipes [5]. Nevertheless, due to the curved geometry of pipe surfaces and limitations in illumination conditions, insufficient depth of field at the edges and severe specular reflections frequently occur, which significantly degrades the recognition accuracy of micro-cracks. Although some studies have introduced 3D point cloud techniques and multimodal fusion strategies to improve detection performance [6,7], these approaches generally involve higher system complexity and increased deployment costs, restricting their large-scale industrial application.

Despite notable progress, high-precision detection of surface defects on stainless steel pipes still faces two key challenges. First, the strong specular reflection characteristics of metallic surfaces often lead to uneven illumination, highlight regions, and shadow areas, which can easily confuse defect regions with background textures. Second, micro-cracks covered by oxide layers or mixed with machining scratches often exhibit highly similar texture characteristics to real defects, making reliable discrimination difficult and limiting the generalization capability of existing models [8,9,10,11].

Convolutional neural networks (CNNs), owing to their powerful automatic feature learning capability, have been widely applied in industrial defect detection tasks [12,13]. Current object detection methods can generally be divided into two-stage approaches (e.g., R-CNN [14], Faster R-CNN [15], Mask R-CNN [16]) and one-stage approaches (e.g., SSD [17] and the YOLO series [18,19,20]). While two-stage methods typically achieve high detection accuracy, their high computational complexity restricts real-time performance. In contrast, one-stage detectors provide a more favorable balance between accuracy and efficiency, among which YOLOv8 has demonstrated strong performance in real-time industrial inspection scenarios.

To systematically address the above challenges, this paper proposes an improved detection algorithm termed YOLOv8s-BISW, which introduces three targeted modifications to the YOLOv8s baseline, each motivated by a specific limitation identified in the current pipeline.

First, the uneven illumination and low contrast caused by factory lighting conditions and metallic surface reflections obscure crack boundaries. Gamma correction with CLAHE is therefore introduced as a preprocessing module to globally adjust brightness and locally enhance contrast, improving the visibility of crack regions before they enter the detection network.

Second, the PAN-FPN neck structure in YOLOv8s fuses multi-scale features through simple concatenation, which treats features from different levels equally and often loses fine-grained spatial details essential for small crack detection. To address this, the Bidirectional Feature Pyramid Network (BiFPN), originally proposed by Tan et al. [21] for efficient multi-scale feature fusion, replaces the PAN-FPN neck. BiFPN introduces learnable fusion weights and cross-scale skip connections, enabling the network to adaptively prioritize features most relevant to crack detection.

Third, strong specular reflection on stainless steel surfaces generates substantial background interference that can mislead the detector. The Spatial Group-wise Enhance (SGE) attention mechanism is embedded into the backbone to strengthen crack-related feature responses within each spatial group while suppressing irrelevant background activations. Unlike channel-wise attention mechanisms that operate globally, SGE preserves spatial specificity, making it more suitable for localizing small, irregular crack targets.

Finally, the Complete IoU (CIoU) loss used in YOLOv8s penalizes aspect ratio deviations based on proportional similarity rather than absolute size differences. For stainless steel pipe cracks—which exhibit aspect ratios ranging from approximately 2:1 to over 15:1 and vary in length from several millimeters to several centimeters—this proportional penalty is insufficient for accurate bounding box regression. The Wise-IoU (WIoU) loss function is adopted to replace CIoU, incorporating a dynamic focusing mechanism that assigns higher regression priority to high-quality predictions while suppressing harmful gradients from low-quality samples [22,23,24].

Since the release of YOLOv8 in January 2023, several subsequent versions have been proposed in the YOLO family, including YOLO-NAS (2023), YOLOv9 (2024), YOLOv10 (2024), YOLO11 (2024), YOLOv12 (2025), and YOLOv13 (2025). YOLOv9 introduced programmable gradient information (PGI) to mitigate the information bottleneck in deep networks; YOLOv10 eliminated the need for non-maximum suppression through consistent dual label assignments; and YOLOv12 incorporated an area attention mechanism to enhance feature representation. Despite these advances, YOLOv8s is chosen as the baseline in this study for three reasons. First, YOLOv8s remains one of the most extensively validated and widely deployed detectors in industrial settings, offering a mature ecosystem and stable inference performance (4.4 ms per image in our experimental setup). Second, the modifications proposed in this work—BiFPN, SGE attention, and WIoU loss—operate on the neck, backbone, and loss function, respectively, and are orthogonal to the backbone-level innovations introduced in later YOLO versions; these modules can be transferred to newer architectures in future work. Third, YOLOv8s provides a favorable accuracy–speed trade-off with relatively low computational requirements, which is essential for real-time deployment on edge devices in factory inspection environments.

The remainder of this paper is organized as follows. Section 2 details each modification and its technical implementation. Section 3 describes the experimental setup and presents ablation studies that isolate the contribution of each component, as well as comparisons with mainstream detectors. Section 4 concludes the paper.

## 2. Methodology

The proposed improvements are developed from three aspects: image quality enhancement, feature representation strengthening, and loss function optimization. First, an image enhancement module is constructed to alleviate the influence of uneven illumination and surface contamination. Second, BiFPN [25] and SGE modules are introduced into the network architecture to enhance multi-scale feature fusion and emphasize defect-related regions. Finally, the WIoU [26] loss function is adopted to improve bounding box regression for irregular crack targets. The effectiveness of the proposed method is systematically validated on the STF dataset.

### 2.1. Image Enhancement Method

In industrial inspection environments, stainless steel pipes are typically imaged under factory lighting conditions that produce uneven illumination across the curved metallic surface. Dark regions obscure fine crack details, while bright regions caused by specular reflection can saturate the sensor, both degrading the quality of input images. Conventional histogram equalization amplifies noise in low-contrast regions and is therefore unsuitable for crack detection where subtle texture differences matter. Due to insufficient illumination, low contrast, and strong specular reflection on stainless steel pipe surfaces, crack regions are often difficult to distinguish from the background. To address this problem, an image enhancement strategy that combines Gamma correction and Contrast Limited Adaptive Histogram Equalization (CLAHE [27,28,29]) is employed.

**Figure 1:**
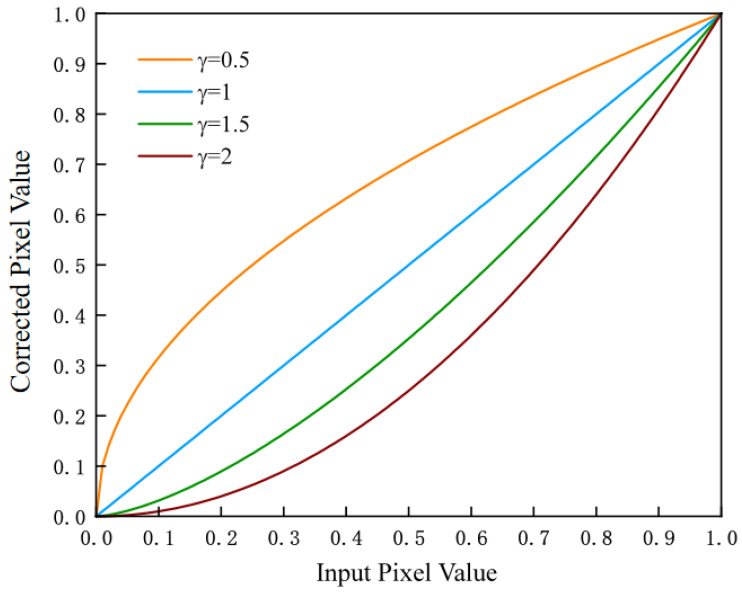
Plot of the Gamma function.

Gamma correction is a nonlinear intensity transformation technique that adjusts the gray-level distribution of an image to improve overall brightness and contrast [30,31,32]. It can be expressed as:

(1) $$s=c\cdot r^{\gamma }$$

where r denotes the original pixel value, s represents the transformed pixel value, c is the brightness coefficient (set to 1 in this study), and γ is the adjustment parameter. The plot of the gamma function is shown in Figure 1. When γ<1, image brightness is enhanced; when γ>1, brightness is reduced. The graph of the gamma function is shown in Figure 1.

Conventional histogram equalization often amplifies local noise. Therefore, CLAHE is adopted to enhance local contrast while effectively suppressing noise amplification, which is particularly beneficial for highlighting crack boundaries. The contrast limiting factor is defined as:

(2) $$\beta =\frac{M}{N}\left[1+\frac{\alpha }{100}\left(S_{\max}-1\right)\right]$$

where M denotes the size of the local window (M = 8 × 8 in this study), N represents the number of gray levels, α is the clipping factor (set to 2.0 in this study), and $S_{max}$ denotes the maximum allowable pixel count in each region.

**Figure 2:**
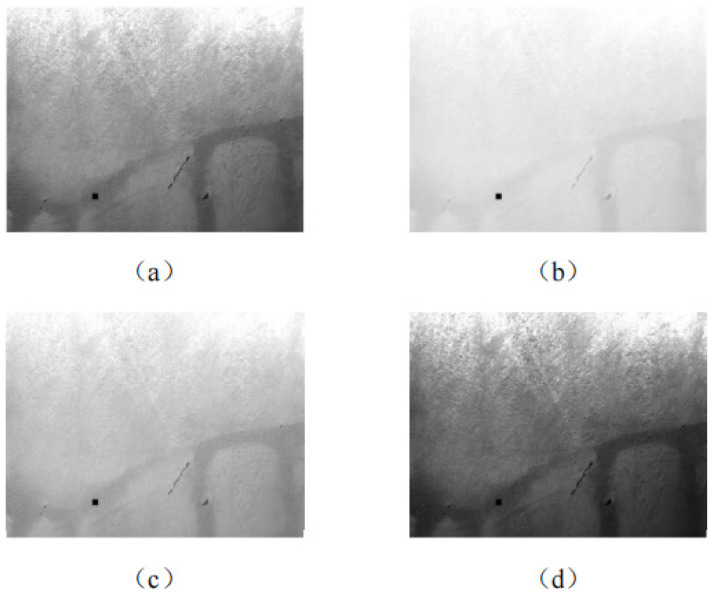
Comparison of crack images under different Gamma correction parameters: (**a**) γ = 0.5, (**b**) γ = 1.0, (**c**) γ = 1.5, (**d**) γ = 2.0.

**Figure 3:**
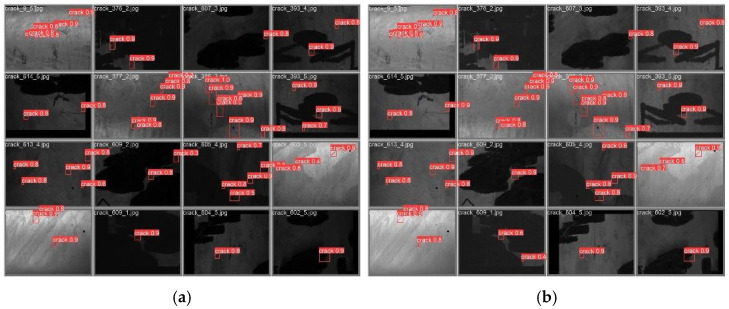
Confidence comparison under different gamma correction parameters. (**a**) Confidence without gamma correction; (**b**) Confidence under gamma correction.

By combining Gamma correction for global brightness adjustment with CLAHE for local contrast enhancement, the proposed preprocessing strategy effectively improves the distinguishability of crack regions under complex background conditions, as illustrated in Figure 2 (γ = 2.0 was adopted in this study), where crack boundaries that are barely discernible under factory lighting become clearly visible after Gamma correction, thereby providing higher-quality inputs for subsequent defect detection. Quantitative evidence of this improvement is presented in Figure 3, where the detection confidence of crack regions increases notably after preprocessing. It should be noted that this image enhancement is applied as an offline, static preprocessing step to the entire dataset before training, rather than being integrated as a dynamic layer within the inference pipeline. Consequently, it does not contribute to the inference latency of the deployed model.

### 2.2. Defect Detection Algorithm Architecture

**Figure 4:**
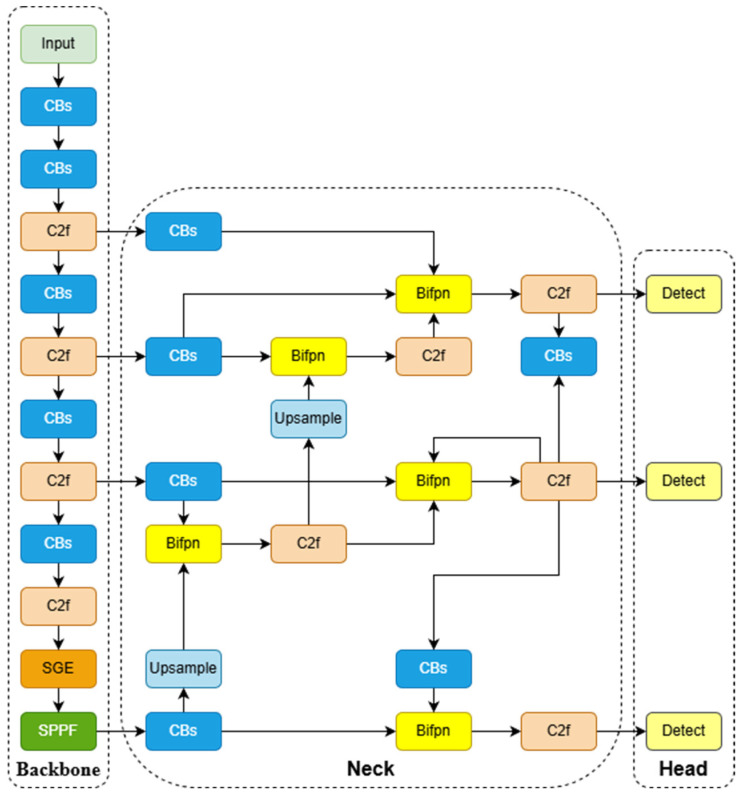
Algorithmic framework of the YOLOv8s-BISW model.

To improve the detection performance of the YOLOv8s model on stainless steel pipe crack defects, an improved algorithm named YOLOv8s-BISW is proposed based on the original YOLOv8s architecture. The overall structure of the proposed model is shown in Figure 4.

Considering the characteristics of crack defects, such as small size, complex shape, and low contrast, three improvements are introduced into the baseline model. First, the SGE attention mechanism is embedded into the backbone network to enhance the feature representation of crack regions and suppress background interference. Second, the original neck structure is replaced with the BiFPN feature fusion network, which strengthens multi-scale feature fusion and improves the detection capability for small targets. Finally, the WIoU loss function is adopted instead of the CIoU loss to further improve the accuracy of bounding box regression.

Through these improvements, the proposed YOLOv8s-BISW model achieves better robustness and detection performance in complex background environments.

### 2.3. Feature Fusion Network

**Figure 5:**
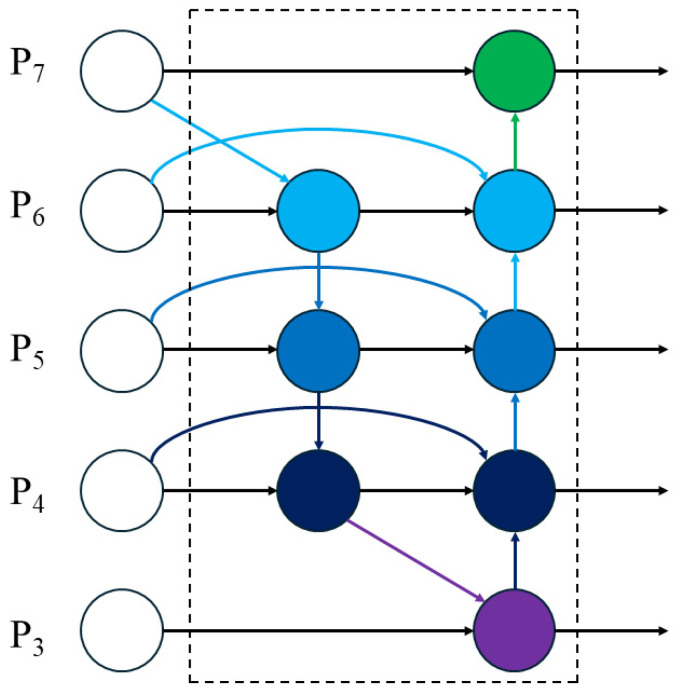
BiFPN structural framework.

The neck network of YOLOv8s adopts a PAN-FPN architecture, which fuses multi-scale features through top-down and bottom-up concatenation. While effective for general object detection, this design has a critical limitation for crack detection on stainless steel pipes: concatenation assigns equal weight to all input features regardless of their relevance to the target. High-level feature maps carry rich semantic information but lose spatial details through repeated downsampling, whereas low-level features preserve fine-grained edge and texture cues essential for localizing small cracks. Under simple concatenation, these spatially precise shallow features are diluted by semantically strong but spatially coarse deep features, increasing the difficulty of detecting small crack targets. Tan et al. identified this limitation and proposed the Bidirectional Feature Pyramid Network (BiFPN), which replaces uniform concatenation with learnable weighted fusion and adds cross-scale skip connections to preserve information flow across all levels. Following this approach, BiFPN is introduced into YOLOv8s-BISW, as shown in Figure 5, to adaptively prioritize the feature scales most relevant to crack detection.

The feature fusion process of BiFPN can be expressed as:

(3) $$O=\sum_{i}\frac{w_{i}}{\epsilon +\sum_{j}w_{j}}\cdot I_{i}$$

where O denotes the output feature map, $I_{i}$ represents the input feature maps at different scales, and $w_{i}$ denotes the corresponding learnable weights. To ensure stability, the weights are constrained to be non-negative by the ReLU function, and ε=0.0001 is used to avoid division by zero.

Through this weighted fusion mechanism, BiFPN can adaptively adjust the contribution of feature maps at different scales, thereby improving the model’s ability to detect crack defects, especially small and low-contrast targets.

### 2.4. Attention Mechanism

**Figure 6:**
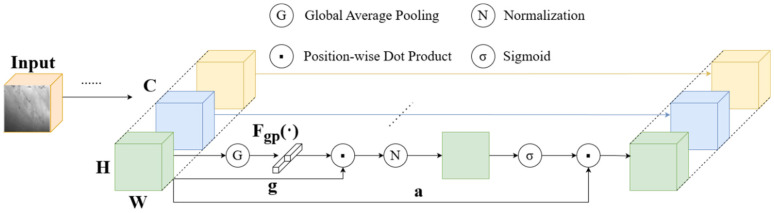
Architecture of the SGE model.

Stainless steel pipe surfaces exhibit strong specular reflection due to their metallic nature, creating high-intensity highlight regions that share visual characteristics with crack edges—both appear as sharp brightness transitions. This similarity causes the detector to produce false positives in non-defect regions. Attention mechanisms can mitigate this by selectively enhancing defect-relevant features. However, channel-wise attention (e.g., SENet) operates globally across the entire spatial extent, which may inadvertently amplify background highlights. Similarly, the Convolutional Block Attention Module (CBAM) combines channel and spatial attention sequentially; however, its spatial attention branch operates at full-map scale and lacks the grouped local modeling capability needed to isolate fine crack features from specular highlights on metallic surfaces. In contrast, the Spatial Group-wise Enhance (SGE) mechanism divides feature maps into groups along the channel dimension and computes spatial attention within each group independently. By modeling the relationships among features within each group, SGE enhances the consistency of semantic information and strengthens discriminative crack features while suppressing background interference. This group-wise design preserves spatial locality and is particularly suitable for crack detection, where defects occupy only a small fraction of the image area under complex backgrounds. The architecture of the SGE model is shown in Figure 6.

The calculation process of the SGE module can be divided into four steps.

(1) Global semantic feature extraction

Given the input feature group $\chi =\{x_{1},x_{2},\ldots ,x_{m}\}$, $x_{i}\in R^{C/G}$, m=H×W, C denotes the number of channels, and G represents the number of groups, spatial global average pooling is applied to obtain the global semantic vector:

(4) $$g=F_{\mathrm{gp}}\left(\chi \right)=\frac{1}{m}\sum_{i=1}^{m}x_{i}$$

(2) Local-global similarity computation:

The similarity between each local feature $x_{i}$ and the global semantic vector g is computed to obtain the importance coefficient:

(5) $$c_{i}=g\cdot x_{i}$$

(3) Coefficient normalization and parameter calibration:

To avoid scale variation among samples, the coefficients are normalized, and two learnable parameters, γ (scaling) and β (shifting), are introduced for affine transformation:

(6) $$\hat{c_{i}}=\frac{c_{i}+\mu_{c}}{\sigma_{c}+\epsilon }$$

(7) $$\mu_{c}=\frac{1}{m}\sum_{j=1}^{m}c_{j}$$

(8) $$\sigma_{c}^{2}=\frac{1}{m}\sum_{j=1}^{m}{\left(c_{j}-\mu_{c}\right)}^{2}$$

(9) $$a_{i}=\gamma \cdot \hat{c_{i}}+\beta$$

where ε=0.0001 is a small constant for numerical stability. The number of parameters γ and β is consistent with the number of groups G (G = 32 was adopted in this study), introducing negligible computational overhead compared with the backbone network.

(4) Feature enhancement:

Finally, the enhanced feature is obtained by applying the Sigmoid activation as a modulation weight:

(10) $$\hat{x_{i}}=x_{i}\cdot \sigma \left(a_{i}\right)$$

**Figure 7:**
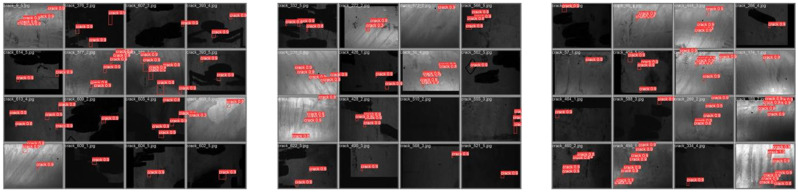
Integrate SGE module recognition results.

The output enhanced feature group is denoted as $\hat{\chi }=\{\hat{x_{1}},\hat{x_{2}},\ldots ,\hat{x_{m}}\}$. Through this mechanism, the SGE module effectively strengthens crack-related feature responses and improves the robustness and stability of the model under complex background conditions. Figure 7 presents a comparative analysis of recognition results with and without the SGE module. In the absence of SGE, the network exhibits dispersed activation across both crack and background regions, particularly in areas affected by specular reflection. After embedding SGE, the activation becomes markedly concentrated on crack regions while background responses are substantially suppressed, confirming the effectiveness of SGE in enhancing defect-related feature representation.

It is instructive to compare SGE with the area attention mechanism recently introduced in YOLOv12. YOLOv12 partitions the feature map into equal-sized spatial regions and applies self-attention with FlashAttention optimization within each region, thereby achieving a broad receptive field at a manageable computational cost. In contrast, SGE operates by grouping features along the channel dimension and applying a lightweight gating mechanism—global average pooling followed by similarity-based sigmoid modulation—within each group. This design introduces only 2 × G additional parameters (where G = 32, representing the number of groups), which is negligible relative to the backbone, whereas area attention requires full query–key–value computations within each spatial partition. For the stainless steel pipe crack detection task, where real-time performance is a strict requirement (the final YOLOv8s-BISW model achieves an inference time of 3.7 ms per image), the minimal computational overhead of SGE makes it a more practical choice for industrial deployment. Nevertheless, integrating the area attention paradigm from YOLOv12 into the proposed framework represents a promising direction for future investigation.

### 2.5. Loss Function Optimization

In object detection tasks, the bounding box regression loss plays a crucial role in determining localization accuracy. The YOLOv8s baseline model adopts the CIoU (Complete Intersection over Union) loss by default, which jointly considers overlap area, center distance, and aspect ratio constraints. However, the aspect ratio constraint in CIoU is mainly based on proportional similarity rather than actual size differences. When the predicted box and the ground truth box share similar aspect ratios, CIoU may produce similar loss values even if their absolute sizes differ significantly. This limitation makes CIoU less suitable for crack targets characterized by large scale variation and irregular shapes. This is especially problematic for the STF dataset, where crack aspect ratios range from below 2:1 to above 15:1. For instance, consider a predicted box of 20 × 4 and a ground-truth box of 200 × 40 (both with aspect ratio 5:1). CIoU assigns the same aspect-ratio penalty of v ≈ 0.62 to both because v depends only on the ratio w/h. In contrast, WIoU produces distinct loss values reflecting their absolute scale differences, thereby assigning higher regression priority to the smaller target where localization precision is more critical.

Moreover, in real inspection scenarios, factors such as uneven illumination and occlusion often lead to distortion in crack appearance, further reducing the robustness of CIoU during regression optimization.

To address these issues, this study introduces the WIoU (Wise-IoU) loss function for bounding box regression optimization. WIoU incorporates a dynamic focusing mechanism and a gradient gain strategy, assigning different regression priorities to prediction samples of varying quality. High-quality predictions are emphasized, while low-quality predictions are suppressed, thereby reducing harmful gradients and improving training stability.

The WIoU loss is defined as:

(11) $$L_{\mathrm{WIoU}}=1-\frac{\sum_{i=1}^{n}w_{i}\cdot L_{\mathrm{IoU}\left(b_{i},g_{i}\right)}}{\sum_{i=1}^{n}w_{i}}$$

where n denotes the number of annotated defects, $b_{i}$ represents the i-th predicted bounding box, $g_{i}$ is the corresponding ground truth box, $L_{IoU}(b_{i},g_{i})$ denotes the IoU loss between the two boxes, and $w_{i}$ is the adaptive weight dynamically adjusted according to the confidence and size of the target.

By introducing the WIoU loss, the proposed model achieves more accurate regression for irregular crack targets, effectively reducing localization errors and improving the stability and reliability of detection results.

## 3. Experiments

### 3.1. Dataset Description

Currently, there is no publicly available benchmark dataset specifically designed for surface crack detection on stainless steel pipes. Therefore, this study constructs a dedicated dataset, termed STF (Steel Tube Flaw), based on images collected from real industrial environments.

**Figure 8:**
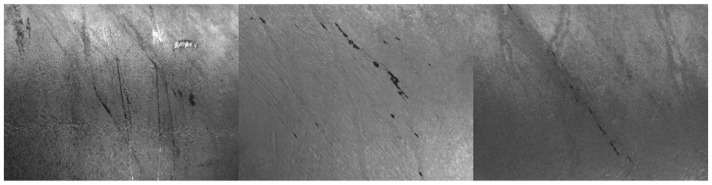
Surface cracks on steel pipes (original resolution: 1922 × 2592).

The images were captured using a Hikvision MV-CL042-91GC line-scan industrial camera. After data cleaning and duplicate removal, a total of 2490 high-quality grayscale images were retained, each with a resolution of 1922 × 2592 pixels. Representative samples of the dataset are shown in Figure 8.

The dataset was divided into a training set (1992 images) and a validation set (498 images) at a ratio of 8:2 to ensure reliable model training and evaluation.

All crack targets were manually annotated using the LabelImg tool. The category was uniformly labeled as “0 (crack)”. Each annotation contains the center coordinates and the normalized width and height of the bounding box. The annotation files were saved in YOLO-format TXT files, corresponding one-to-one with the image data, and were used for model training and performance validation.

However, it should be noted that the current study does not employ an independent hold-out test set, and the validation set may have been used for model selection, which could lead to an overestimation of the model’s generalization capability. Future studies should validate the model’s generalization capability on strictly separate hold-out data to further confirm these findings.

### 3.2. Experimental Setup and Parameter Settings

#### 3.2.1. Hardware and Software Configuration

**Figure 9:**
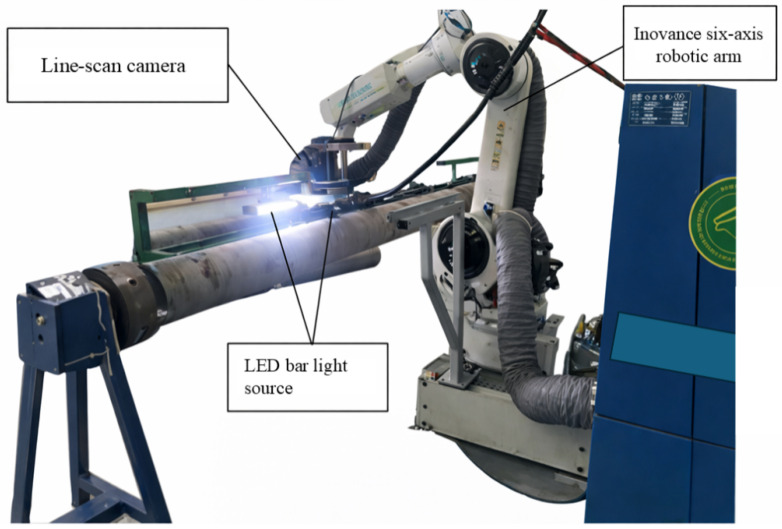
Setup of the field experimental equipment.

The experimental hardware platform includes a rotating servo motor and a seventh-axis moving motor (Inovance MS1H3-18C15CD), as well as a PC workstation (Windows 10 operating system, Intel Core i5-12490F CPU, NVIDIA GeForce RTX 4070 Ti SUPER GPU, and 32 GB memory). The software environment is based on Python 3.8, and the PyTorch 2.0 framework is used for model training and inference. The construction of the on-site experimental equipment is shown in Figure 9.

#### 3.2.2. Training Parameter Settings

The experimental parameters are set as follows: the number of training epochs is 300, the initial learning rate is 0.01, the batch size is 16, the bounding box loss gain is 7.5, and the IoU threshold is 0.7. The optimizer is stochastic gradient descent (SGD), with a momentum of 0.9 and a weight decay of 0.0005, to ensure sufficient model convergence while avoiding overfitting.

### 3.3. Evaluation Metrics

To objectively evaluate the performance of the proposed model on stainless steel pipe crack detection, several widely used metrics in object detection are adopted, including Precision, Recall, and mean Average Precision (mAP).

First, the Intersection over Union (IoU) is used to measure the overlap between the predicted bounding box and the ground truth box, which is defined as:

(12) $$IoU=\frac{S_{pred}\cap S_{gt}}{S_{pred}\cup S_{gt}}$$

where $S_{pred}$ denotes the predicted bounding box area and $S_{gt}$ denotes the ground truth area.

The Precision and Recall are calculated as follows:

(13) $$P=\frac{TP}{TP+FP}$$

(14) $$R=\frac{TP}{TP+FN}$$

where TP (True Positive) represents correctly detected crack samples, FP (False Positive) represents incorrectly detected samples, and FN (False Negative) represents missed detections.

The Average Precision (AP) is obtained by integrating the Precision–Recall curve, which reflects the comprehensive detection performance under different confidence thresholds. The mean Average Precision (mAP) is calculated by averaging the AP values over all categories:

(15) $$mAP=\frac{1}{N}\sum_{i=1}^{N}AP_{i}$$

where N denotes the number of categories. Since this study focuses on single-class crack detection, the mAP value is equivalent to the AP of this category.

These evaluation metrics provide a comprehensive assessment of the proposed method in terms of detection accuracy and completeness.

### 3.4. Ablation Experiments

#### 3.4.1. Design of Ablation Experiments

Ablation experiments are a fundamental method in deep learning research for verifying the effectiveness of different modules. Inspired by the concept of “ablation” in medical research, this approach is widely used in tasks such as object detection.

To verify the effectiveness of each proposed component in the YOLOv8s-BISW algorithm, the original YOLOv8s model (V8-0) was selected as the baseline. Seven groups of ablation models were designed to analyze the impact of BiFPN, SGE, and WIoU when applied individually or in combination. The model configurations are shown in Table 1.

#### 3.4.2. Analysis of Ablation Results

The ablation results are presented in Table 2. It can be observed that each proposed module contributes positively to the overall performance of the model.

(1) Effect of Single Modules:

BiFPN only (V8-1): Precision, Recall, and mAP50 increased from 0.913, 0.960, and 0.946 (baseline) to 0.921, 0.970, and 0.964, respectively. This demonstrates that BiFPN effectively enhances multi-scale feature fusion, enabling better aggregation of global context and local crack details and alleviating the problem of insufficient feature extraction for small and low-contrast targets.

SGE only (V8-2): The mAP50 increased to 0.960. Although Precision slightly decreased to 0.901, Recall remained high at 0.970. This indicates that the SGE attention mechanism enhances the detection of ambiguous cracks by strengthening crack-related features and suppressing background noise, thereby improving sensitivity under complex imaging conditions.

WIoU only (V8-3): The mAP50 increased to 0.955, with only minor fluctuations in Precision and Recall. This suggests that WIoU improves bounding box regression by better adapting to the irregular shapes of surface cracks, thereby enhancing localization accuracy.

(2) Effect of Module Combinations:

BiFPN + WIoU (V8-4): The mAP50 reached 0.974, outperforming the single-module versions of BiFPN (0.964) and WIoU (0.955). This indicates a complementary effect: BiFPN provides higher-quality features, while WIoU further refines localization accuracy.

SGE + WIoU (V8-5): The mAP50 achieved 0.978, representing the second-best performance among all models. This combination forms an effective “detection–localization” loop, where SGE enhances crack sensitivity and WIoU improves positioning accuracy.

BiFPN + SGE (V8-6): The mAP50 reached 0.977, which is very close to that of V8-5. BiFPN provides richer multi-scale features for SGE, allowing the attention mechanism to focus more accurately on crack regions and thereby improving robustness under complex backgrounds.

**Figure 10:**
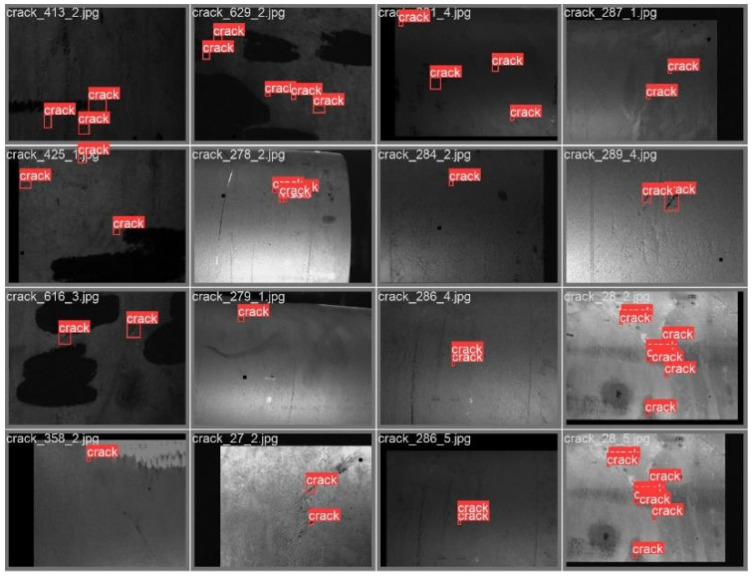
Detection results of the YOLOv8s-BISW model.

(3) Full Module Integration:

The fully integrated model, YOLOv8s-BISW (V8-7), achieved the best overall performance, with a Precision of 0.920, a Recall of 0.970, and an mAP50 of 0.979. This result confirms that the combination of BiFPN feature fusion, SGE attention enhancement, and WIoU loss optimization forms a mutually complementary strategy that comprehensively improves both recognition accuracy and localization capability. The detection results of the final model are shown in Figure 10.

### 3.5. Comparison with Other Object Detection Algorithms

To further verify the effectiveness of the proposed improved YOLOv8 model, comparative experiments were conducted on the same dataset against five mainstream object detection algorithms. The compared models include the single-stage detectors SSD, YOLOv3, YOLOv5, and YOLOv8, as well as the two-stage detector Faster R-CNN. The performance comparison results are shown in Table 3, where T denotes the results obtained using the Gamma + CLAHE enhanced dataset (applied identically to all compared models for fairness).

As shown in Table 3, the original YOLOv8 achieved an mAP of 0.926 with a detection time of 4.4 ms without data enhancement. Among all comparison models, YOLOv8 trained with data enhancement (T-YOLOv8) achieved the best overall performance, with an mAP of 0.946 and a detection time of only 3.7 ms.

In comparison, the enhanced versions of YOLOv3 (T-YOLOv3) and YOLOv5 (T-YOLOv5) achieved mAP values of 0.933 and 0.935, respectively, with detection times of 8.5 ms and 14.9 ms, both significantly slower than YOLOv8.

For the other models, T-SSD obtained an mAP of 0.776 with a detection time of 13.2 ms, while T-Faster R-CNN achieved only 0.695 mAP with a detection time of 6.8 ms. These results indicate that both SSD and Faster R-CNN perform inferior to the YOLO series in terms of both accuracy and efficiency.

Overall, the improved YOLOv8 model demonstrates superior performance in both detection accuracy and inference speed compared with other mainstream detection algorithms, especially when combined with data enhancement. These results further validate the effectiveness of the proposed improvement strategy in enhancing crack detection performance.

## 4. Conclusions

In response to the harsh production environment of stainless steel pipes, the high labor intensity of manual inspection, and the limitations of existing techniques, such as excessive false detections, high missed-detection rates, and long inspection time, this study proposes a novel detection algorithm, YOLOv8s-BISW, by improving the YOLOv8s model to achieve efficient and accurate detection of surface defects on stainless steel pipes.

The main contributions of this work are summarized as follows. First, a combined Gamma correction and CLAHE-based image enhancement module is introduced to effectively alleviate uneven illumination and blurred defect imaging. Second, the SGE attention module is embedded into the network to dynamically enhance defect-related features while suppressing background interference, thereby improving classification and localization accuracy. Third, the BiFPN structure is adopted to optimize multi-scale feature fusion, enhancing the model’s adaptability to cracks of different sizes. Fourth, the WIoU loss function is employed to replace CIoU, improving bounding box regression performance and reducing localization errors for irregular defects.

To verify the effectiveness of the proposed method, an industrial experimental platform was constructed, and the STF stainless steel pipe crack dataset was collected and established. Through ablation experiments and comparisons with mainstream detection algorithms, the results demonstrate that the proposed YOLOv8s-BISW model achieves an mAP of 0.979, which is 0.033 higher than the original YOLOv8s, while maintaining a detection time of only 3.7 ms per image, thus achieving a favorable balance between accuracy and real-time performance. Compared with algorithms such as SSD and Faster R-CNN, the proposed method shows clear advantages in both accuracy and speed. However, it should be noted that the current study does not employ an independent hold-out test set, and the validation set may have been used for model selection, which could lead to an overestimation of the model’s generalization capability. Future studies should validate the model’s generalization capability on strictly separate hold-out data to further confirm these findings.

The application of this algorithm can significantly reduce labor intensity, improve production efficiency, and lower safety risks caused by missed defect detection, providing strong technical support for technological upgrading and safe production in the stainless steel pipe manufacturing industry.

Future work will focus on three directions. First, further lightweight optimization of the model will be explored through network pruning, quantization, and compression techniques to reduce computational cost and enable deployment on industrial edge devices. Additionally, inference latency benchmarking on representative edge devices (e.g., NVIDIA Jetson Xavier NX/Orin Nano) will be conducted to evaluate real-world deployability. Second, the generalization capability of the model will be enhanced by investigating generative data augmentation and multimodal data fusion strategies, integrating information from ultrasound and infrared imaging to overcome the limitations of single visual inspection and improve adaptability to different materials and working conditions. Third, the application scope will be expanded by extending the proposed method to other industrial defect detection scenarios, such as bearings and weld seams, and by integrating real-time image processing and feedback mechanisms to enable automatic crack depth estimation and adaptive control, thereby further promoting the large-scale application of intelligent inspection technologies in manufacturing. Fourth, the reported results are derived from single training runs; future work should conduct multiple runs with different random seeds and report the mean ± standard deviation to provide a more robust statistical characterization of the model’s performance.

## Figures and Tables

**Table 1:** Ablation study model design.

Model	Model Design
V8-1	Only BiFPN feature fusion structure is introduced
V8-2	Only the SGE attention module is embedded in the backbone network
V8-3	Only WIoU is used to replace the CIoU loss function
V8-4	WIoU is introduced based on V8-1
V8-5	WIoU is introduced based on V8-2
V8-6	SGE is embedded based on V8-1
V8-7	BiFPN, SGE, and WIoU are integrated (i.e., YOLOv8s-BISW)

**Table 2:** Ablation study results.

Baseline	Bifpn	SGE	WIoU	P	R	mAP50
V8-0				0.913	0.960	0.946
V8-1	✓			0.921	0.970	0.964
V8-2		✓		0.901	0.970	0.960
V8-3			✓	0.901	0.960	0.955
V8-4	✓		✓	0.920	0.930	0.974
V8-5		✓	✓	0.911	0.940	0.978
V8-6	✓	✓		0.917	0.970	0.977
V8-7	✓	✓	✓	0.920	0.970	0.979

**Table 3:** Algorithm comparison (T denotes models trained with the Gamma + CLAHE enhanced dataset).

Baseline	mAP	Detection Time/ms
Yolov8	0.926	4.4
T-Yolov3	0.933	8.5
T-Yolov5	0.935	14.9
T-SSD	0.776	13.2
T-Faster-rcnn	0.695	6.8
T-Yolov8	0.946	3.7
YOLOv8s-BISW	0.979	3.7

## Data Availability

The raw STF dataset code supporting the conclusions of this article will be made available by the corresponding author on reasonable request.
